# Impact of white matter hyperintensity volumes estimated by automated methods using deep learning on stroke outcomes in small vessel occlusion stroke

**DOI:** 10.3389/fnagi.2024.1399457

**Published:** 2024-06-21

**Authors:** Minwoo Lee, Chong Hyun Suh, Jong-Hee Sohn, Chulho Kim, Sang-Won Han, Joo Hye Sung, Kyung-Ho Yu, Jae-Sung Lim, Sang-Hwa Lee

**Affiliations:** ^1^Department of Neurology, Hallym University Sacred Heart Hospital, Hallym University College of Medicine, Anyang, Republic of Korea; ^2^Department of Radiology and Research Institute of Radiology, Asan Medical Center, University of Ulsan College of Medicine, Seoul, Republic of Korea; ^3^Department of Neurology, Chuncheon Sacred Heart Hospital, Hallym University College of Medicine, Chuncheon, Republic of Korea; ^4^Institute of New Frontier Research Team, Hallym University, Chuncheon, Republic of Korea; ^5^Department of Neurology, Asan Medical Center, University of Ulsan College of Medicine, Seoul, Republic of Korea

**Keywords:** white matter hyperintensity, small vessel disease, SVO stroke, stroke outcome, machine learning

## Abstract

**Introduction:**

Although white matter hyperintensity (WMH) shares similar vascular risk and pathology with small vessel occlusion (SVO) stroke, there were few studies to evaluate the impact of the burden of WMH volume on early and delayed stroke outcomes in SVO stroke.

**Materials and methods:**

Using a multicenter registry database, we enrolled SVO stroke patients between August 2013 and November 2022. The WMH volume was estimated by automated methods using deep learning (VUNO Med-DeepBrain, Seoul, South Korea), which was a commercially available segmentation model. After propensity score matching (PSM), we evaluated the impact of WMH volume on early neurological deterioration (END) and poor functional outcomes at 3-month modified Ranking Scale (mRS), defined as mRS score >2 at 3 months, after an SVO stroke.

**Results:**

Among 1,718 SVO stroke cases, the prevalence of subjects with severe WMH (Fazekas score ≥ 3) was 68.9%. After PSM, END and poor functional outcomes at 3-month mRS (mRS > 2) were higher in the severe WMH group (END: 6.9 vs. 13.5%, *p* < 0.001; 3-month mRS > 2: 11.4 vs. 24.7%, *p* < 0.001). The logistic regression analysis using the PSM cohort showed that total WMH volume increased the risk of END [odd ratio [OR], 95% confidence interval [CI]; 1.01, 1.00–1.02, *p* = 0.048] and 3-month mRS > 2 (OR, 95% CI; 1.02, 1.01–1.03, *p* < 0.001). Deep WMH was associated with both END and 3-month mRS > 2, but periventricular WMH was associated with 3-month mRS > 2 only.

**Conclusion:**

This study used automated methods using a deep learning segmentation model to assess the impact of WMH burden on outcomes in SVO stroke. Our findings emphasize the significance of WMH burden in SVO stroke prognosis, encouraging tailored interventions for better patient care.

## Introduction

Small-vessel occlusion (SVO) stroke is a distinct type of stroke characterized by small, deep cerebral infarcts primarily involving the perforating arteries (Rost et al., 2010a). While SVO stroke is generally associated with a better prognosis than large-vessel stroke, patients with this subtype nevertheless show considerable variability in outcomes (Ntaios et al., 2016). Therefore, understanding the factors that influence stroke outcomes in SVO stroke is critical to optimizing patient management and improving the long-term prognosis.

One potential factor influencing stroke outcomes is the burden of white matter hyperintensities (WMH), defined as the extent of white matter pathology in the brain (Kim and Lee, 2015). The white matter is composed of myelinated nerve fibers that facilitate efficient communication between different brain regions, and it has been hypothesized that the WMH burden on magnetic resonance imaging (MRI) may contribute to worse outcomes in patients with SVO stroke. WMH and SVO stroke are consequences of endothelial failure, which share several vascular risk factors that may explain their coexistence (Wen and Sachdev, 2004; Wardlaw et al., 2013a).

Several studies have indicated a higher prevalence and severity of white matter disease in patients with SVO stroke, suggesting an association between the WMH burden and this type of stroke (Yan et al., 2015; Zhang et al., 2015; Helenius et al., 2017; Tian et al., 2022). However, to date, no studies have examined how the severity of WMH burden in the deep and periventricular areas impacts outcomes following acute SVO stroke. Previous studies have further shown that WMH burden may worsen stroke outcomes in large-vessel occlusion stroke and cryptogenic stroke (Jeong et al., 2018; Griessenauer et al., 2020; Derraz et al., 2022). Given the hypothesis of an association between WMH burden severity and the prevalence of SVO stroke, it is plausible that the effect of WMH burden may worsen outcomes following acute SVO stroke.

The widely used Fazekas scale is an easy and quick method used in clinical practice to estimate the volume of WMHs (Fazekas et al., 1987). However, due to the inherent heterogeneity in the size, number, shape, and location of WMH, a more reliable and objective method to assess WMH volume may be needed (Vermeer et al., 2003; Ding et al., 2020). In recent years, automated methods using deep learning, such as convolutional neural networks, have been applied to measure WMH volumes, with varying results (Jiang et al., 2020; Balakrishnan et al., 2021). One commercially available segmentation model, the VUNO Med-DeepBrain (Suh et al., 2020), focuses on treating highly imbalanced WMH labels, especially, primarily deep WMH, by applying generalized cube loss (Joo et al., 2022).

This study aimed to investigate the impact of the severity of WMH burden, estimated using a deep learning-based automatic WMH segmentation algorithm, on stroke outcome in acute SVO stroke, a field that has not yet been evaluated.

## Methods

### Study population

Patients with acute ischemic stroke were consecutively enrolled in two university-affiliated multicenter registry databases between August 2013 and November 2022. We identified patients with acute ischemic stroke due to SVO according to the Trial of Org 10172 in Acute Stroke Treatment (TOAST) classification, with some modifications (Ko et al., 2014). Accordingly, we reviewed all diffusion-weighted imaging (DWI) lesions in enrolled patients to confirm SVO stroke. SVO lesions were documented as a single lesion with the largest diameter of ≤ 20 mm on an axial slice of DWI that penetrate the arterial infarction of the basal ganglia, corona radiata, thalamus, or pons without a cardioembolic source or relevant stenosis of the corresponding artery. We excluded patients meeting the following criteria: (1) those with an unavailable initial MRI, (2) those unavailable for early neurological deterioration (END) and a 3-month modified Rankin Scale (mRS) assessment, and (3) those with a pre-stroke mRS score ≥2.

### Data collection and definition of parameters

Demographic, clinical, laboratory, and outcome data were obtained directly from the web-based registry databases of the two institutions. We prospectively recruited patients from Hallym Sacred Heart Hospital, a regional cardiocerebrovascular center in southwestern Gyeonggi-do, South Korea, and Chuncheon Sacred Heart Hospital, a regional emergency center in western Gangwon-do, South Korea. These hospitals serve populations of ~1 million and 500,000, respectively. Both institutions are part of the Clinical Research Collaboration for Stroke in Korea (CRCS-K) registry, in which prospectively trained stroke coordinators enter information about stroke patients, including their outcomes, into the registry website using the same enrollment protocol (Bae et al., 2022).

We identified WMH as an MRI feature of cerebral small vessel disease (SVD), according to the Standards for Reporting Vascular Changes on Neuroimaging (STRIVE) criteria (Wardlaw et al., 2013b; Duering et al., 2023). Baseline WMH assessment was performed by two expert vascular neurologists (M Lee and S-H Lee) in a double-blinded manner [interclass correlation coefficient [ICC] = 0.88, *p* < 0.001]. The degree of WMH burden was rated visually on axial FLuid-Attenuated Inversion Recovery (FLAIR) images using the modified Fazekas scale and was graded separately as periventricular WMH (PWMH) or deep WMH (DWMH; Vermeer et al., 2003). A total Fazekas score, ranging from 0 to 6, was estimated by summing the Fazekas scores for PWMH and DWMH. We defined severe WMH as a total Fazekas score of ≥3, as previously described (Guo et al., 2021). In accordance with the STRIVE criteria (Staals et al., 2014; Duering et al., 2023), the SVD score was determined by a double-blinded review of FLAIR images by two expert vascular neurologists (M Lee and S-H Lee; ICC = 0.92, *p* < 0.001).

Acute SVO stroke was defined as a single lesion with the largest diameter of ≤ 20 mm in an axial slice of DWI for penetrating artery infarction of the basal ganglia, corona radiata, thalamus, or pons based on the TOAST classification (Ay et al., 2005; Rost et al., 2010a; Ko et al., 2014). In addition, this lesion should be matched to a low signal on apparent diffusion coefficient (ADC) maps and correlate with the neurological symptom (Rost et al., 2010a).

Brain MRI was performed on a 3.0 T superconducting system (3T Achieva X-series; Philips Healthcare, Best, Netherlands). All brain MRI protocols have been described in Supplementary Table 1.

### Outcome measures

In this study, the primary outcome measure was END, defined as an increase of at least 1 point in motor power or a worsening of the total National Institute of Health Stroke Scale (NIHSS) score of ≥2 points within 72 h of hospitalization, as compared with the initial NIHSS score (Park et al., 2020). The secondary outcome measure was poor functional outcomes at 3 months, defined as an mRS score of >2.

### Quantitative WMH volume assessment

We performed WMH segmentation using a pre-trained model based on the 2D UNet architecture with a ResNet34 encoder (Joo et al., 2022). The model features an encoder-decoder framework with skip connections, where the encoder pulls out features from the input while the decoder generates a segmentation mask based on the latent feature vector. Skip connections facilitate improved learning of high-level features by concatenating the outputs of the encoder extracts with their corresponding decoder inputs. The workflow of the 2D UNet architecture with the ResNet34 encoder is presented in Supplementary Figure 1.

Resizing each input T2-FLAIR MRI image to a constant dimension is necessary to construct the training dataset; however, we found a certain amount of distortion when the 2D MRI image was resized to a predetermined size. This is because the number of slices varies, meaning that the image size is adjusted to the specified dimensions (256, 256, z) when the original dimensions (x, y, z) and voxel sizes (a, b, c) are given in the RAS (Right, Anterior, Superior) orientation, voxel size is adjusted to (a ^*^ 256/x, b ^*^ 256/x, c), and voxel intensity is normalized within the range of [0, 255] using min-max normalization. All images used for training and testing were normalized using min-max scaling within the range of [0, 255] to maintain a uniform voxel intensity distribution. We subsequently applied the T2-FLAIR intracranial volume segmentation tool available in the software to perform brain extraction and reduce artifacts (Suh et al., 2023). The training dataset did not include any acute or old infarcts. Small infarcts were included in WMH because they appear high on FLAIR. Acute infarcts that do not appear frequently on FLAIR imaging were inevitably omitted. PWMH and DWMH were segmented separately. The PWMH boundary was located 1 cm from the ventricular wall. The reliability and validation of the automated WMH segmentation and Fazekas scale prediction model of VUNO Med-DeepBrain demonstrated its accuracy compared with manual annotation in a previous study (Jung et al., 2022). Furthermore, we intend to continue accumulating evidence to support its reliability.

### Statistical analysis

We hypothesized that increased WMH volume would increase the risk of END and poor functional outcomes in patients with acute SVO stroke. We separated the groups into the following subgroups: normal to mild WMH (Fazekas score, 0–2) and severe WMH (Fazekas score, 3–6). Covariates and outcomes between the two groups were compared using Pearson's chi-squared test for categorical variables and the Student's *t*-test or the Mann-Whitney *U*-test for continuous variables.

To account for potential covariate imbalances and confounding factors between the normal-to-mild and severe WMH groups, we applied propensity score matching (PSM) techniques to enhance the robustness of our analysis. The propensity score for each group was defined as the probability of being in the normal-to-mild WMH group, based on the patients' baseline demographics and vascular risk factors in the baseline logistic regression analysis. Using these propensity scores, we then matched the normal-to-mild and severe WMH groups in a 1:1 ratio using the nearest-neighbor method. The covariates used in PSM analysis included all of the demographic variables, stroke risk factors, treatment modalities, lesion locations, and SVD markers that have been demonstrated to influence the outcome, as described in Table 1. Logistic regression analysis was performed on the PSM cohort to assess the effect of WMH volume on outcomes. Specifically, we used quantitative measures of total WMH, PWMH, and DWMH volume as independent variables to evaluate their associations with post-stroke outcomes. Furthermore, we adjusted for significant covariates with a *p*-value < 0.05 that were deemed clinically plausible in relation to outcomes. We subsequently performed the Hosmer-Lemeshow test to confirm the goodness of fit of the logistic regression model. Statistical analyses were performed using Statistical Package for the Social Sciences (SPSS) version 21.0 software (IBM Corporation, Armonk, NY, USA) and R version 4.0.3 moonBook and MatchIt (R Core Team 2020, R Foundation for Statistical Computing, Vienna, Austria).

**Table 1:** Baseline characteristics between the normal to mild WMH group and the severe WMH group in total and PSM cohort.

	**Total cohort**			**PSM cohort**		
	**Normal-to-mild WMH**	**Severe WMH**	***p*-value**	**Normal-to-mild WMH**	**Severe WMH**	***p*-value**
	***n* = 534**	***n* = 1,184**		***n* = 534**	***n* = 534**	
Age, year (SD)^†^	62.1 (11.4)	68.5 (12.3)	0.003	62.1 (11.4)	76.1 (9.9)	< 0.001
Male sex, *n* (%)^*^	353 (66.1)	685 (57.6)	0.001	353 (66.1)	242 (45.3)	< 0.001
Initial NIHSS, score (IQR)^‡^	2 (1–3)	2 (1–4)	0.004	2 (1–3)	3 (1–5)	< 0.001
Prior stroke, *n* (%)^*^	66 (12.4)	276 (23.3)	< 0.001	66 (12.4)	213 (39.9)	< 0.001
Hypertension, *n* (%)^*^	284 (53.2)	715 (60.2)	0.01	284 (53.2)	372 (69.7)	< 0.001
Diabetes mellitus, *n* (%)^*^	177 (33.1)	388 (32.8)	0.91	177 (33.1)	162 (30.3)	0.36
Hyperlipidemia, *n* (%)^*^	109 (20.4)	314 (26.5)	0.01	109 (20.4)	183 (34.3)	< 0.001
Current smoking, *n* (%)^*^	157 (29.4)	257 (21.7)	0.001	157 (29.4)	60 (11.2)	< 0.001
Prior antithrombotic, *n* (%)^*^	121 (22.7)	328 (27.7)	0.03	121 (22.7)	181 (33.9)	< 0.001
Prior statin, *n* (%)^*^	91 (17.0)	232 (19.6)	0.23	91 (17.0)	95 (17.8)	0.81
Prior IVT, *n* (%)^*^	34 (6.4)	45 (4.7)	0.16	34 (6.4)	19 (3.6)	0.059
Lesion location, *n* (%)^*^			0.10			0.58
Anterior	287 (53.7)	687 (58.0)		287 (53.7)	297 (55.6)	
Posterior	247 (46.3)	497 (42.0)		247 (46.3)	237 (44.4)	
**SVD markers**, ***n*** (%)^*^						
MBs	73 (13.7)	230 (19.4)	0.004	73 (13.7)	143 (26.8)	< 0.001
ePVS	10 (1.9)	124 (10.5)	< 0.001	10 (1.9)	124 (23.2)	< 0.001
Lacune	80 (15.0)	304 (25.7)	< 0.001	80 (15.0)	217 (40.6)	< 0.001
**Total SVD score**, ***n*** (%)^*^						
0	385 (72.1)	152 (12.8)		385 (72.1)	51 (9.6)	
1	132 (24.7)	631 (53.3)		132 (24.7)	202 (37.8)	
2	17 (3.2)	247 (20.9)		17 (3.2)	150 (28.1)	
3	0 (0.0)	122 (10.3)		0 (0.0)	99 (18.5)	
4	0 (0.0)	32 (10.3)		0 (0.0)	32 (6.0)	

WMH, white matter hyperintensity; PSM, propensity score matching; SD, standard deviation; NIHSS, National Institute of Health Stroke Scale; IQR, interquartile range; IVT, intravenous thrombolysis; SVD, small vessel disease; MBs, microbleeds; ePVS, extensive perivascular space; PWMH, periventricular WMH; DWMH, deep WMH.

^*^Calculated using the chi-square test.

^†^Calculated using the Student's t-test.

^‡^Calculated using the Mann–Whitney U-test.

## Results

**Figure 1:**
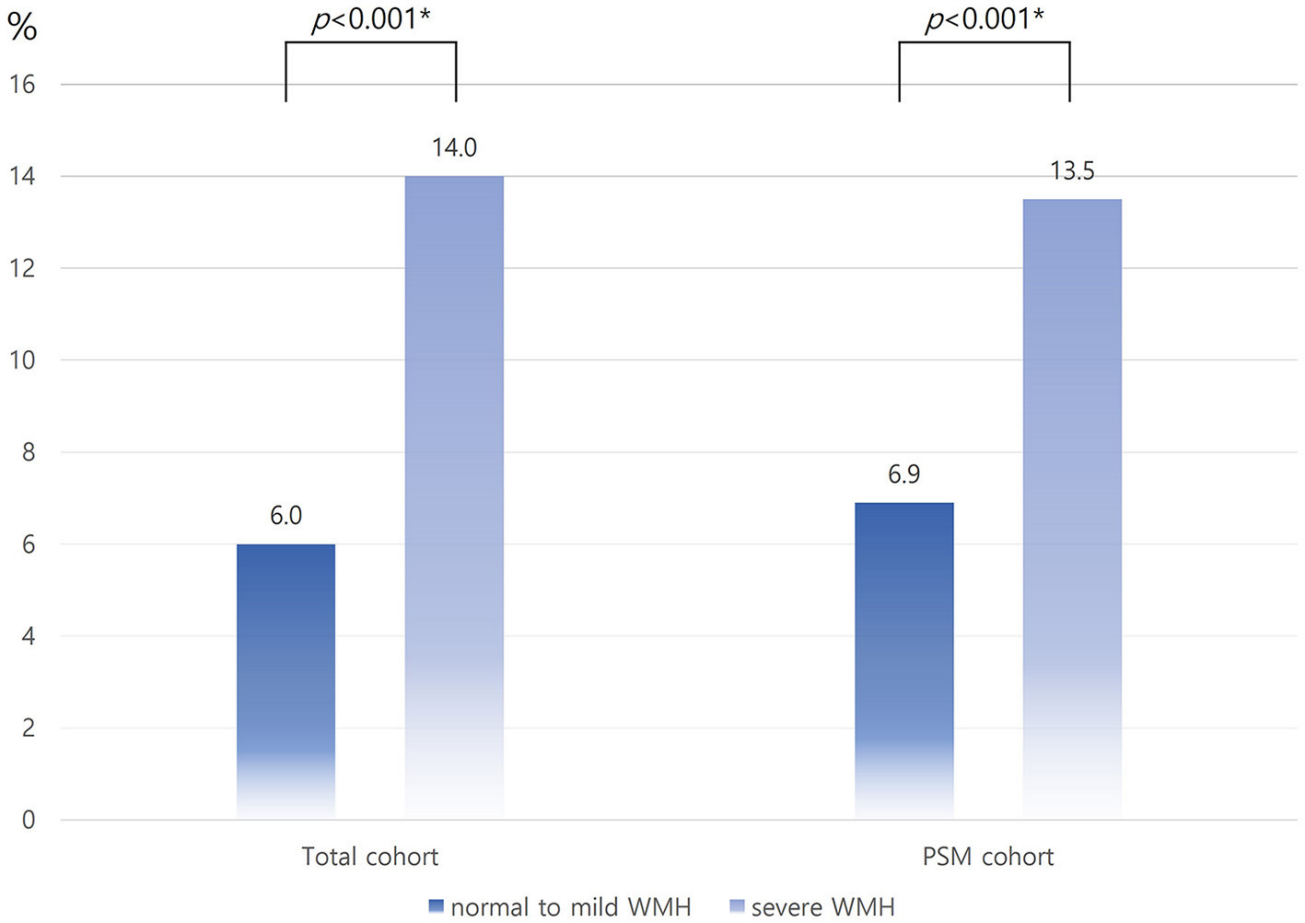
Distribution of quantitative WMH volume according to the Fazekas scale in the PSM cohort. WMH, white matter hyperintensity; PSM, propensity score matching; P-Fazekas, periventricular Fazekas scale; D-Fazekas, Deep Fazekas scale. *Using the linear contrast test.

Of the 7,782 consecutive patients with acute ischemic stroke treated in the two centers during the study period, 1,792 were diagnosed with acute SVO stroke. Of these patients with SVO strokes, 1,718 who met the inclusion criteria were enrolled in this study. Among the enrolled patients, 68.9% (1,184/1,718) were classified into the severe WMH group. Patients with severe WMH burden were generally older; had severe neurological symptoms; had a higher prevalence of prior stroke, hypertension, and hyperlipidemia; were less likely to belong to male sex and current smokers; and were more likely to have previously received antithrombotic therapy. Higher total SVD scores (3 and 4), as defined by the STRIVE criteria, were more common in the severe WMH group. Compared to the normal-to-mild WMH group, the quantitative total WMM, DWMH, and PWMH volumes were higher in the severe WMH group (Table 1). The mean quantitative WMH volume was likely to be increased with categories of Fazekas scales (PWMH volume: mean ± SD, 8.49 ± 5.97 in Fazekas scale 0–1; mean ± SD, 15.63 ± 10.59 in Fazekas scale 2; mean ± SD, 22.08 ± 11.10 in Fazekas scale 3; DWMH volume: mean ± SD, 3.30 ± 3.90 in Fazekas scale 0–1; mean ± SD, 10.99 ± 12.28 in Fazekas scale 2; mean ± SD, 15.57 ± 18.51 in Fazekas scale 3, Figure 1). After PSM, 534 patients with a normal-to-mild WMH burden were matched in a 1:1 ratio with those with a severe WMH burden. In the PSM cohort, the baseline characteristics of the two groups were similar and balanced (Table 1).

**Figure 2:**
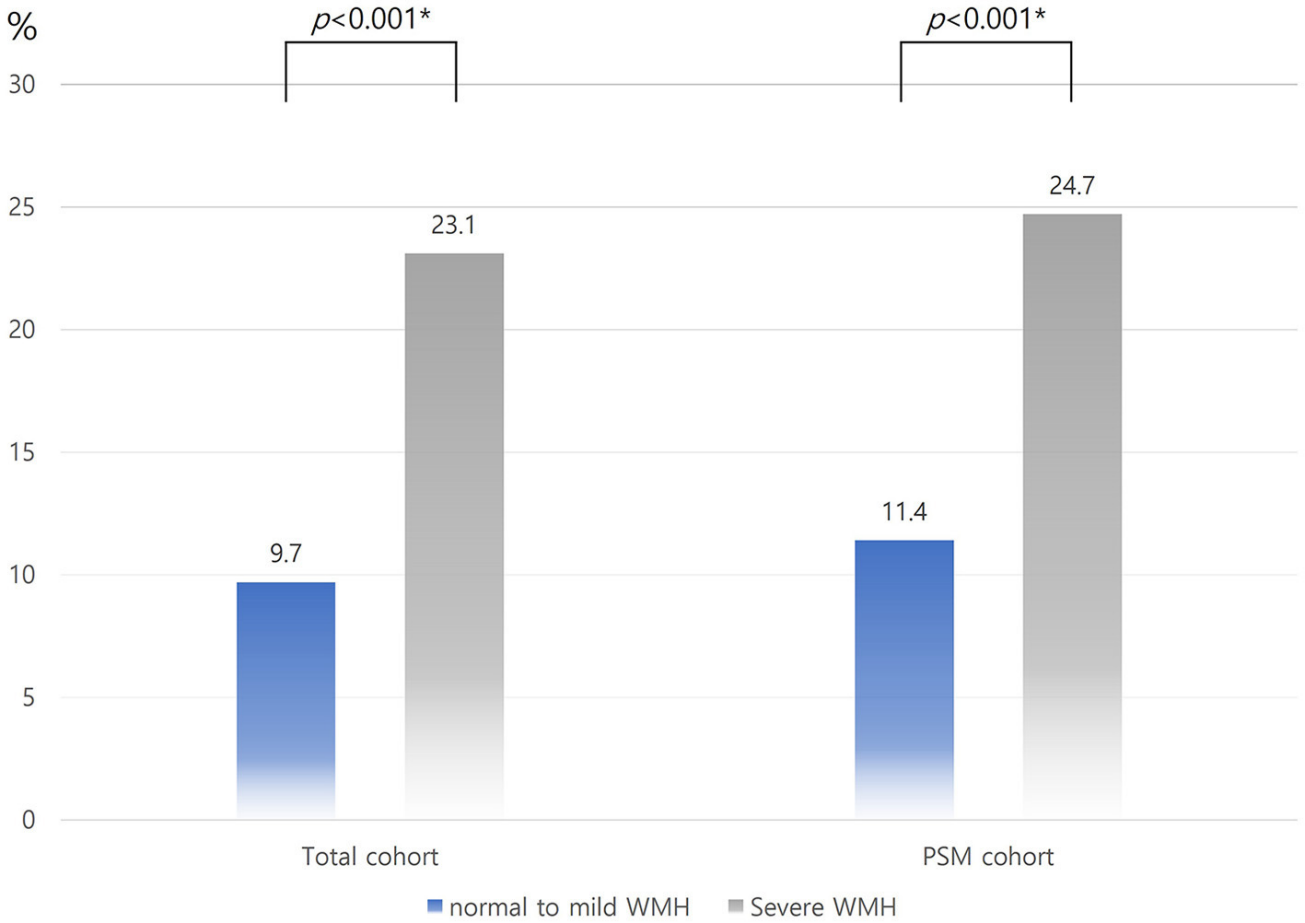
Distribution of END according to WMH burden in the total and PSM cohorts. END, early neurologic deterioration; WMH, white matter hyperintensity; PSM, propensity score matching. *Using the chi-square test.

**Figure 3:**
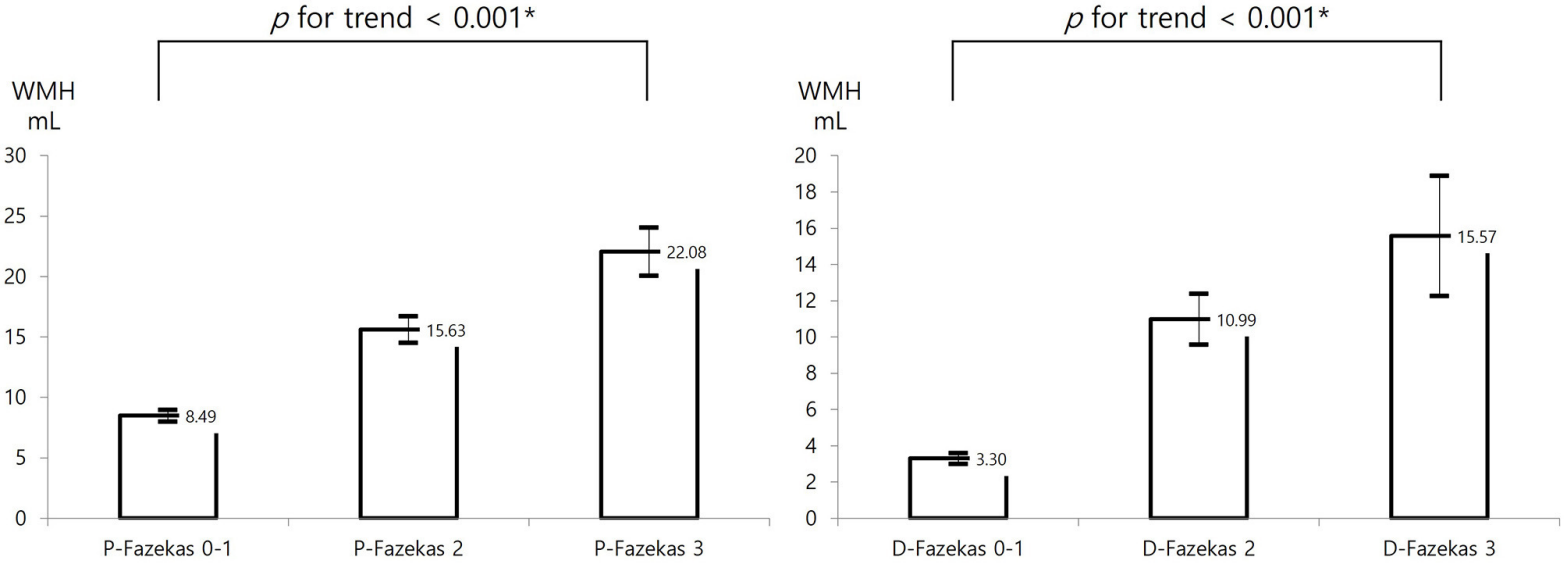
Distribution of 3-month mRS > 2 according to WMH burden in the total and PSM cohorts. mRS, modified Rankin Scale; WMH, white matter hyperintensity; PSM, propensity score matching. *Using the chi-square test.

In the entire cohort, the occurrence of END was higher in the severe WMH group than in the normal-to-mild WMH group (14.0 vs. 6.0%, *p* < 0.001); this effect remains significant in the PSM cohort (13.5 vs. 6.9%, *p* < 0.001; Figure 2). The proportion of patients with poor functional outcomes (mRS > 2) at 3 months was higher in the severe WMH group than in the normal-to-mild WMH group in the total cohort (23.1 vs. 9.7%, *p* < 0.001), as well as in the PSM cohort (24.7 vs. 11.4%, *p* < 0.001, Figure 3).

In this study, the covariates were imbalanced between the two groups, despite PSM. Therefore, we conducted a Hosmer-Lemeshow test to assess the goodness of fit of the logistic regression model in the PSM cohort. A logistic regression analysis in the PSM cohort showed that the total quantitative WMH volume increased the risk of END (OR, 1.01; 95% CI, 1.00–1.02; *p* = 0.048) and 3-month mRS > 2 (OR, 1.02; 95% CI, 1.01–1.03; *p* < 0.001). Furthermore, in logistic regression analysis in the PSM cohort, the quantitative DWMH volume was found to be associated with an increased risk of both END and 3-month mRS > 2 (END: OR, 1.02; 95% CI, 1.01–1.04; *p* = 0.01; 3-month mRS > 2: OR, 1.04; 95% CI, 1.02–1.06; *p* < 0.001, Table 2, Supplementary Tables 2, 3). In addition, quantitative PWMH was associated with 3-month mRS > 2 in the logistic regression analysis (OR, 1.03; 95% CI, 1.01–1.05; *p* = 0.01), but not with END in the PSM cohort (Table 2, Supplementary Tables 2, 3). The logistic regression analysis of the total cohort further revealed that the effect of the WMH volume on stroke outcomes remained unchanged (Supplementary Tables 4, 5). The Hosmer-Lemeshow test indicated a good logistic model fit for this study. For END, the total WMH volume had a chi-square test value of 3.06 with a significance level of *p* = 0.93, and the PWMH volume had a chi-square test value of 7.28 with a significance level of *p* = 0.51. The chi-square test result for the DWMH volume was 5.31 with a significance level of *p* = 0.72, while that for total WMH volume with a 3-month mRS > 2 was 5.19 with a significance level of *p* = 0.74. Meanwhile, the chi-square test result for PWMH volume was 4.39 with a significance level of *p* = 0.82 and that for DWMH volume was 4.41 with a significance level of *p* = 0.82.

**Table 2:** Effect of WMH burden volumes per 1 ml on stroke outcomes in SVO stroke using the PSM cohort.

	**END**			**3-month mRS** > **2**		
	**OR**	**95% CI**	***p*-value**	**OR**	**95% CI**	***p*-value**
Total WMH volume	1.01	1.00–1.02	0.048	1.02	1.01–1.03	< 0.001
PWMH volume	1.01	0.99–1.03	0.43	1.03	1.01–1.05	0.01
DWMH volume	1.02	1.01–1.04	0.008	1.04	1.02–1.06	< 0.001

WMH, white matter hyperintensity; SVO, small vessel occlusion; PSM, propensity score matching; END, early neurologic deterioration; mRS, modified Rankin Scale; PWMH, periventricular WMH; DWMH, deep WMH.

Adjusted for age, sex, initial NIHSS, prior stroke, hypertension (HTN), hyperlipidemia (HL), current smoking, prior antithrombotic use, and total SVD score.

## Discussion

The purpose of this study was to investigate the impact of WMH burden, as quantified using a deep-learning-based automated segmentation algorithm, on stroke outcomes in patients with acute SVO stroke. This study revealed several important findings that contribute to our understanding of the relationship between WMH burden and stroke outcomes.

Overall, the results of this study provide compelling evidence that an increased WMH burden is associated with worse stroke outcomes in patients with acute SVO stroke. The primary outcome measure, END, was consistently more common in patients with a high WMH burden, both in the overall cohort and after adjusting for potential confounders by PSM. This finding is consistent with previous studies, which showed an association between WMH and worse outcomes in various stroke subtypes (Rost et al., 2010a,b). However, one previous study did not identify a significant correlation between WMH and END in patients with SVO stroke (Ryu et al., 2017). Overall, our findings suggest a more differentiated role for WMHs: DWMHs are more closely associated with END, whereas PWMHs appear to influence long-term functional outcomes. This distinction is critical, as it highlights the importance of considering the spatial distribution of WMHs when assessing their potential impact on stroke recovery. By incorporating the results of a previous study into our discussion, we acknowledge the broader consensus on the significance of WMH volume while providing additional insights into the regional effects that may guide more targeted interventions in the management of SVO stroke. As such, this study extends this knowledge to SVO stroke and highlights the importance of WMH burden as a predictor of END. The secondary outcome measure, poor functional outcomes at 3 months, showed a similar pattern. Patients with severe WMH burden had a higher proportion of poor functional outcomes, highlighting the potential long-term implications of WMH burden in SVO stroke. This finding is consistent with those of previous studies, which have shown an association between WMH burden and poor functional outcomes and increased disability after stroke. Thus, the current study further supports the notion that the WMH burden has a broad impact on post-stroke recovery and overall quality of life.

In light of these findings, several mechanisms underlying the association between the WMH burden and stroke outcomes in patients with SVO stroke warrant further discussion. WMH and SVO strokes share common vascular risk factors and underlying pathophysiological mechanisms, including endothelial dysfunction and microvascular impairment (Wen and Sachdev, 2004; Wardlaw et al., 2013a). The presence of extensive WMH may reflect a more widespread disruption of the microvascular network, which could lead to impaired perfusion, compromised tissue integrity, and limited potential for neurovascular recovery following an ischemic insult (Fernando et al., 2006; Chen et al., 2021). Furthermore, WMH burden may indicate a higher susceptibility to subsequent ischemic events, leading to a compounding effect on stroke severity and recovery.

Interestingly, the present study revealed different associations between DWMH and PWMH and different stroke outcomes, providing valuable insights into the nuanced effects of white matter pathology on post-stroke recovery. The observed association between quantitative DWMH volume and an increased risk of END is a significant finding. DWMH represents white matter changes that occur deeper in the brain, often reflecting chronic ischemic damage to the small penetrating arteries supplying this region (Cai et al., 2022). This result suggests that structural integrity and connectivity within critical neural networks may be compromised in patients with a higher DWMH burden (Moody et al., 2004; Porcu et al., 2020). The disrupted white matter tracts may contribute to the END and delayed functional status observed in these patients, potentially by amplifying the effects of the initial ischemic insult and impairing neural compensation mechanisms. In contrast, the association between the quantitative PWMH volume and delayed functional outcomes, as opposed to END, highlights a different aspect of white matter pathology. PWMH is typically associated with small-vessel disease in superficial white matter regions close to the ventricles. This area is vulnerable to changes in the blood-brain barrier permeability and cerebral fluid dynamics (van den Heuvel et al., 2006; Iliff et al., 2012). The correlation between PWMH and 3-month mRS score suggests that these periventricular changes may influence the long-term recovery trajectories rather than immediate neurological deterioration. It is plausible that the impact of PWMH on functional outcomes may be related to its disruptive effects on neural networks, which are critical for higher-order cognitive and motor functions (Goulay et al., 2020). These differential associations between DWMH, PWMH, and stroke outcomes highlight the need for tailored approaches to stroke management and prognosis. END is a critical event that can significantly influence patient management decisions, warranting vigilant monitoring and early intervention in patients with a higher DWMH burden. In contrast, the delayed functional outcomes associated with PWMH highlight the importance of addressing white matter pathology to optimize long-term recovery. Identifying patients with a substantial PWMH burden could help design strategies to support cognitive rehabilitation and address potential cognitive impairments that may develop over time.

In the present study, we performed a quantitative analysis of WMH volume using a deep-learning-based segmentation algorithm, which provided a more accurate and objective assessment than traditional visual grading scales, such as the Fazekas scale. We further proposed the mean quantitative volume as PWMH and DWMH using deep learning, in accordance with the categories of the Fazekas scale. Previous studies using automatic segmentation showed the cutoff values of the WMH total quantitative volume and Fazekas scale scores (Andere et al., 2022; Joo et al., 2022). This methodological advancement is significant because the WMH burden is known to exhibit considerable variability in terms of size, shape, and location. The automated segmentation model used in this study, which was based on the UNet architecture with a ResNet34 encoder, adds to the growing body of literature using deep-learning techniques to address these challenges in a reliable and reproducible manner.

Nevertheless, this study has several limitations that should be considered when interpreting the results. First, despite the use of PSM to control for potential confounders, residual confounding variables may have influenced the observed associations. Second, this study focused on a specific stroke subtype (SVO), and the results may therefore not be directly applicable to other stroke subtypes. Third, although the deep learning-based segmentation algorithm represents a more advanced approach for quantifying the WMH burden, validation of the accuracy and generalizability of the algorithm is essential. Fourth, the study was conducted exclusively on Korean individuals, thus precluding any generalizations regarding the impact of WMH volume on the study outcome based on race. Nevertheless, a recent study demonstrated no differences in WMH injury after adjusting for various cardiovascular risk factors, even in the presence of racial differences (Austin et al., 2022). Future studies should investigate the effects of the WMH volume on stroke outcomes in other ethnic groups. Finally, the rationale behind the omission of WMHs/intracranial volumes (ICVs) in our study was that the objective of intracranial volume segmentation was to facilitate skull stripping, which in turn would allow for skull artifact correction. In the future, it may be desirable to perform ICV correction on the data from this part of the study.

## Conclusion

In conclusion, this study highlights the detrimental impact of WMH burden on stroke outcomes in patients with acute SVO. These results provide valuable insights into the prognostic significance of WMH burden and further suggest that incorporating quantitative measures of WMH volume into clinical practice could improve risk stratification and guide treatment decisions. However, further research in larger and more diverse cohorts is needed to validate these findings and explore the underlying mechanisms linking WMH burden to stroke outcomes. Eventually, a better understanding of the relationship between WMH and stroke may lead to more targeted interventions to optimize patient management and improve the long-term prognosis in individuals with SVO stroke.

## Data availability statement

The raw data supporting the conclusions of this article will be made available by the authors, without undue reservation.

## Ethics statement

The studies involving humans were approved by Chuncheon Sacred Heart Hospital IRB No. 2023-07-007 and Hallym University Sacred Heart Hospital IRB No. 2021-03-008. The studies were conducted in accordance with the local legislation and institutional requirements. The participants provided their written informed consent to participate in this study.

## Author contributions

ML: Writing – review & editing, Writing – original draft, Investigation, Formal analysis, Conceptualization. CS: Writing – review & editing, Writing – original draft, Methodology, Conceptualization. J-HS: Writing – review & editing, Data curation. CK: Writing – review & editing, Data curation. S-WH: Writing – review & editing, Data curation. JS: Writing – review & editing, Data curation. K-HY: Writing – review & editing, Data curation. J-SL: Writing – review & editing, Data curation. S-HL: Writing – review & editing, Writing – original draft, Supervision, Conceptualization.
