# Supplementary material for: Impact of white matter hyperintensity volumes estimated by automated methods using deep learning on stroke outcomes in small vessel occlusion stroke
Source: Front Aging Neurosci. 2024 Jun 21;16:1399457. doi: 10.3389/fnagi.2024.1399457 (PMC11224430; doi:10.3389/fnagi.2024.1399457)

Supplementary Table 1. Brain MRI scan protocols in this study.

|  | Field of View | Slice thickness (mm) | TR (ms) | TE (ms) |
| --- | --- | --- | --- | --- |
| T1WI | 512ⅹ512 | 5 | 488 | 10 |
| FLAIR | 528ⅹ528 | 5 | 9000 | 120 |
| DWI | 256ⅹ256 | 3 | 3774 | 73 |

Supplementary Table 2. Effect of WMH burden volumes per 1mL on END in SVO stroke using PSM cohort.

|  | OR | 95% CI | p-value |  | OR | 95% CI | p-value |  | OR | 95% CI | p-value |
| --- | --- | --- | --- | --- | --- | --- | --- | --- | --- | --- | --- |
| Total WMH | 1.01 | 1.00-1.02 | 0.048 | PWMH | 1.01 | 0.99-1.03 | 0.43 | DWMH | 1.02 | 1.01-1.04 | 0.008 |
| Age | 1.02 | 0.998-1.04 | 0.08 | Age | 1.02 | 0.999-1.04 | 0.07 | Age | 1.02 | 0.998-1.04 | 0.08 |
| Male | 1.02 | 0.59-1.54 | 0.92 | Male | 1.03 | 0.68-1.54 | 0.91 | Male | 1.02 | 0.68-1.54 | 0.92 |
| Initial NIHSS | 1.10 | 1.03-1.17 | 0.003 | Initial NIHSS | 1.10 | 1.03-1.17 | 0.003 | Initial NIHSS | 1.10 | 1.04-1.18 | 0.002 |
| Prior stroke | 1.96 | 1.26-3.05 | 0.003 | Prior stroke | 1.93 | 1.25-3.00 | 0.003 | Prior stroke | 1.99 | 1.28-3.09 | 0.002 |
| HTN | 0.94 | 0.62-1.42 | 0.77 | HTN | 0.94 | 0.62-1.41 | 0.75 | HTN | 0.95 | 0.63-1.43 | 0.80 |
| HL | 1.33 | 0.89-1.99 | 0.16 | HL | 1.31 | 0.88-1.96 | 0.19 | HL | 1.33 | 0.89-1.99 | 0.17 |
| current smoking | 0.72 | 0.39-1.34 | 0.30 | c smoking | 1.72 | 0.39-1.34 | 0.3 | c smoking | 0.72 | 0.38-1.34 | 0.29 |
| Prior antithrom | 1.12 | 0.72-1.75 | 0.62 | Prior antithrom | 1.12 | 0.72-1.74 | 0.62 | Prior antithrom | 1.11 | 0.71-1.73 | 0.64 |
| Total SVD score |  |  |  |  |  |  |  |  |  |  |  |
| 0 | ref |  |  | 0 | ref |  |  | 0 | ref |  |  |
| 1 | 1.69 | 0.98-2.91 | 0.06 | 1 | 1.75 | 1.02-3.01 | 0.04 | 1 | 1.67 | 0.97-2.88 | 0.07 |
| 2 | 2.20 | 1.17-4.12 | 0.01 | 2 | 2.52 | 1.35-4.73 | 0.004 | 2 | 2.16 | 1.17-4.00 | 0.01 |
| 3 | 1.24 | 0.53-2.91 | 0.63 | 3 | 1.68 | 0.75-3.78 | 0.21 | 3 | 1.08 | 0.46-2.55 | 0.85 |
| 4 | 1.56 | 0.47-5.16 | 0.47 | 4 | 2.48 | 0.82-7.49 | 0.11 | 4 | 1.20 | 0.35-4.10 | 0.77 |

Supplementary Table 3. Effect of WMH burden volumes per 1mL 3-month mRS >2 in SVO stroke using PSM cohort.

|  | OR | 95% CI | p-value |  | OR | 95% CI | p-value |  | OR | 95% CI | p-value |
| --- | --- | --- | --- | --- | --- | --- | --- | --- | --- | --- | --- |
| Total WMH | 1.02 | 1.01-1.03 | <0.001 | PWMH | 1.03 | 1.01-1.05 | 0.01 | DWMH | 1.04 | 1.02-1.06 | <0.001 |
| Age | 1.06 | 1.04-1.08 | <0.001 | Age | 1.06 | 1.04-1.08 | <0.001 | Age | 1.06 | 1.04-1.08 | <0.001 |
| Male | 0.96 | 0.66-1.39 | 0.82 | Male | 0.97 | 0.66-1.40 | 0.85 | Male | 0.96 | 0.66-1.39 | 0.81 |
| Initial NIHSS | 1.46 | 1.35-1.57 | <0.001 | Initial NIHSS | 1.45 | 1.35-1.55 | <0.001 | Initial NIHSS | 1.46 | 1.36-1.57 | <0.001 |
| Prior stroke | 1.61 | 1.07-2.44 | 0.02 | Prior stroke | 1.57 | 1.04-2.36 | 0.03 | Prior stroke | 1.63 | 1.08-2.48 | 0.02 |
| HTN | 0.77 | 0.53-1.12 | 0.17 | HTN | 0.77 | 0.53-1.11 | 0.16 | HTN | 0.78 | 0.54-1.14 | 0.21 |
| HL | 1.37 | 0.94-1.99 | 0.11 | HL | 1.35 | 0.93-1.97 | 0.11 | HL | 1.36 | 0.93-1.98 | 0.11 |
| current smoking | 1.07 | 0.62-1.84 | 0.82 | current smoking | 1.06 | 0.62-1.82 | 0.83 | current smoking | 1.07 | 0.62-1.84 | 0.82 |
| Prior antithrom | 1.11 | 0.73-1.67 | 0.63 | Prior antithrom | 1.11 | 0.74-1.67 | 0.62 | Prior antithrom | 1.08 | 0.72-1.64 | 0.71 |
| Total SVD score |  |  |  | Total SVD score |  |  |  | Total SVD score |  |  |  |
| 0 | ref |  |  | 0 | ref |  |  | 0 | ref |  |  |
| 1 | 1.54 | 0.97-2.46 | 0.07 | 1 | 1.61 | 1.01-2.57 | 0.04 | 1 | 1.54 | 0.96-2.46 | 0.07 |
| 2 | 1.64 | 0.93-2.88 | 0.09 | 2 | 1.92 | 1.10-3.36 | 0.02 | 2 | 1.71 | 0.98-2.96 | 0.06 |
| 3 | 0.96 | 0.45-2.07 | 0.92 | 3 | 1.46 | 0.72-2.98 | 0.3 | 3 | 0.87 | 0.40-1.88 | 0.72 |
| 4 | 1.24 | 0.41-3.73 | 0.70 | 4 | 2.47 | 0.92-6.67 | 0.07 | 4 | 0.95 | 0.30-3.02 | 0.93 |

Supplementary table 4. Effect of WMH burden volumes per 1mL on END in SVO stroke using total cohort.

|  | OR | 95% CI | p-value |  | OR | 95% CI | p-value |  | OR | 95% CI | p-value |
| --- | --- | --- | --- | --- | --- | --- | --- | --- | --- | --- | --- |
| Total WMH | 1.01 | 1.001-1.02 | 0.04 | PWMH | 1.01 | 0.99-1.02 | 0.56 | DWMH | 1.02 | 1.01-1.04 | 0.006 |
| Age | 1.02 | 1.003-1.03 | 0.02 | Age | 1.02 | 1.004-1.03 | 0.01 | Age | 1.02 | 1.003-1.03 | 0.02 |
| Male | 0.884 | 0.60-1.18 | 0.31 | Male | 0.84 | 0.60-1.19 | 0.33 | Male | 0.84 | 0.60-1.18 | 0.32 |
| Initial NIHSS | 1.11 | 1.05-1.17 | <0.001 | Initial NIHSS | 1.11 | 1.05-1.17 | <0.001 | Initial NIHSS | 1.11 | 1.05-1.18 | <0.001 |
| Prior stroke | 2.14 | 1.47-3.10 | <0.001 | Prior stroke | 2.1 | 1.45-3.05 | <0.001 | Prior stroke | 2.16 | 1.49-3.13 | <0.001 |
| HTN | 0.94 | 0.67-1.31 | 0.7 | HTN | 0.93 | 0.67-1.31 | 0.68 | HTN | 0.94 | 0.67-1.32 | 0.73 |
| DM | 0.93 | 0.66-1.31 | 0.69 | DM | 0.94 | 0.67-1.32 | 0.71 | DM | 0.93 | 0.66-1.30 | 0.67 |
| HL | 1.27 | 0.89-1.79 | 0.19 | HL | 1.25 | 0.88-1.77 | 0.21 | HL | 1.26 | 0.89-1.79 | 0.19 |
| current smoking | 0.95 | 0.61-1.47 | 0.8 | current smoking | 0.94 | 0.60-1.46 | 0.77 | current smoking | 0.94 | 0.60-1.47 | 0.79 |
| Prior antithrom | 0.99 | 0.68-1.44 | 0.95 | Prior antithrom | 0.99 | 0.68-1.45 | 0.97 | Prior antithrom | 0.99 | 0.68-1.44 | 0.95 |
| Total SVD score |  |  |  | Total SVD score |  |  |  | Total SVD score |  |  |  |
| 0 | ref |  |  | 0 | ref |  |  | 0 | ref |  |  |
| 1 | 1.67 | 1.09-2.56 | 0.02 | 1 | 1.73 | 1.13-0.65 | 0.01 | 1 | 1.64 | 1.07-2.52 | 0.02 |
| 2 | 1.8 | 1.05-3.08 | 0.03 | 2 | 2.05 | 1.20-3.49 | 0.01 | 2 | 1.74 | 1.03-2.94 | 0.04 |
| 3 | 1.32 | 0.62-2.78 | 0.47 | 3 | 1.71 | 0.84-3.50 | 0.14 | 3 | 1.13 | 0.53-2.40 | 0.76 |
| 4 | 1.75 | 0.57-5.40 | 0.33 | 4 | 2.64 | 0.92-7.54 | 0.07 | 4 | 1.28 | 0.40-4.10 | 0.68 |

Supplementary Table 5. Effect of WMH burden volumes per 1mL 3-month mRS >2 in SVO stroke using total cohort.

|  | OR | 95% CI | p-value |  | OR | 95% CI | p-value |  | OR | 95% CI | p-value |
| --- | --- | --- | --- | --- | --- | --- | --- | --- | --- | --- | --- |
| Total WMH | 1.03 | 1.02-1.03 | <0.001 | PWMH | 1.03 | 1.02-1.05 | <0.001 | DWMH | 1.05 | 1.03-1.06 | <0.001 |
| Age | 1.05 | 1.04-1.07 | <0.001 | Age | 1.06 | 1.04-1.07 | <0.001 | Age | 1.05 | 1.04-1.07 | <0.001 |
| Male | 0.92 | 0.67-1.25 | 0.59 | Male | 0.92 | 0.67-1.36 | 0.59 | Male | 0.93 | 0.68-1.27 | 0.64 |
| Initial NIHSS | 1.49 | 1.40-1.59 | <0.001 | Initial NIHSS | 1.48 | 1.39-1.58 | <0.001 | Initial NIHSS | 1.50 | 1.40-1.59 | <0.001 |
| Prior stroke | 1.80 | 1.26-2.57 | 0.001 | Prior stroke | 1.74 | 1.23-2.48 | 0.002 | Prior stroke | 1.80 | 1.26-2.57 | 0.01 |
| HTN | 0.76 | 0.55-1.03 | 0.08 | HTN | 0.75 | 0.55-1.02 | 0.07 | HTN | 0.77 | 0.56-1.04 | 0.09 |
| DM | 1.33 | 0.98-1.81 | 0.07 | DM | 1.34 | 0.99-1.882 | 0.06 | DM | 1.33 | 0.98-1.81 | 0.07 |
| HL | 1.34 | 0.97-1.86 | 0.08 | HL | 1.34 | 0.97-1.86 | 0.08 | HL | 1.31 | 0.94-1.81 | 0.11 |
| current smoking | 0.93 | 0.62-1.41 | 0.74 | current smoking | 0.93 | 0.62-1.40 | 0.74 | current smoking | 0.92 | 0.61-1.38 | 0.67 |
| Prior antithrom | 0.99 | 0.70-1.41 | 0.97 | Prior antithrom | 0.998 | 0.71-1.41 | 0.99 | Prior antithrom | 0.99 | 0.70-1.40 | 0.96 |
| Total SVD score |  |  |  | Total SVD score |  |  |  | Total SVD score |  |  |  |
| 0 | ref |  |  | 0 | ref |  |  | 0 | ref |  |  |
| 1 | 1.39 | 0.96-2.02 | 0.08 | 1 | 1.46 | 1.01-2.12 | 0.045 | 1 | 1.41 | 0.97-2.04 | 0.07 |
| 2 | 1.59 | 0.98-2.57 | 0.06 | 2 | 1.81 | 1.12-2.91 | 0.02 | 2 | 1.73 | 1.08-2.76 | 0.02 |
| 3 | 0.76 | 0.38-1.52 | 0.92 | 3 | 1.13 | 0.59-2.14 | 0.71 | 3 | 0.73 | 0.36-1.47 | 0.38 |
| 4 | 0.95 | 0.33-2.70 | 0.92 | 4 | 1.93 | 0.75-1.97 | 0.18 | 4 | 0.76 | 0.25-2.29 | 0.62 |

Supplementary figure 1. Working flow diagram of 2D UNet architecture with a ResNet34 encoder.


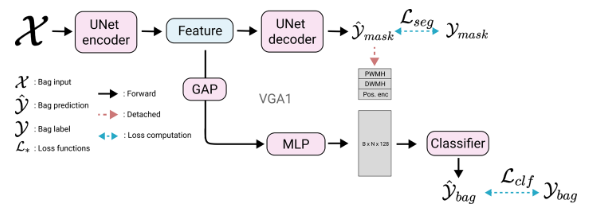

Supplement: Supplementary file 1 [file Data_Sheet_1.docx]
